# Participants in an online weight loss program can improve diet quality during weight loss: a randomized controlled trial

**DOI:** 10.1186/1475-2891-13-82

**Published:** 2014-08-09

**Authors:** Kate M O’Brien, Melinda J Hutchesson, Megan Jensen, Philip Morgan, Robin Callister, Clare E Collins

**Affiliations:** University of Newcastle, 2308 Callaghan, NSW Australia; Priority Research Centre in Physical Activity and Nutrition, University of Newcastle, 2308 Callaghan, NSW Australia

**Keywords:** Diet quality, Index, Score, Weight loss, Intervention

## Abstract

**Background:**

Better diet quality has been associated with less weight gain over time. However, few studies have examined the role of diet quality during weight loss. This study aimed to compare changes in diet quality in overweight/obese adults during a weight loss intervention, and determine whether an association between diet quality score and weight loss exists.

**Methods:**

Overweight or obese (BMI 25-40 kg/m^2^) adults, aged 18–60 years, were recruited from the Hunter Region of NSW, Australia and randomized to one of three groups: a standard online weight loss program (n = 94); an enhanced version of this online program that provided additional personalized feedback and reminders (n = 98); or a wait-list control group (n = 97). Diet quality was calculated using the Australian Recommended Food Score (ARFS) with dietary data from the Australian Eating Survey (AES) Food Frequency Questionnaire at baseline and 12-weeks.

**Results:**

The basic and enhanced groups lost significantly more weight than the control group after 12 weeks (basic -2.2 ± 3.4 kg, enhanced -3.0 ± 4.0 kg, control 0.4 ± 2.4 kg, *P* < 0.001) with no difference between the basic and enhanced groups. The mean change in ARFS in the enhanced group (2.2 ± 5.7) was significantly higher (*P* = 0.03) than the control group. There were no significant differences in change in ARFS between the enhanced and basic, or basic and control groups. The ARFS and the fruit, meat, wholegrain, dairy and water sub-scale scores at 12 weeks were significantly associated with greater weight loss (*P* < 0.05).

**Conclusions:**

Diet quality improved significantly in the enhanced group compared to controls following 12-weeks intervention. Furthermore, higher diet quality was associated with greater weight loss.

**Trial Registration:**

ACTRN12610000197033.

## Background

Obesity is a major public health problem that has reached epidemic proportions worldwide [1]. In Australia, the prevalence of overweight and obesity has dramatically increased over the past two decades [2]. In the 2011–12 National Health Survey, 70% of adult men and 56% of adult women were assessed as being overweight or obese [3]. Excess body weight increases chronic disease risk, including type 2 diabetes, gall bladder disease, heart disease and some cancers, such as cancer of the kidney, colon, and breast [4]. As a result, the prevention of excess weight gain has become a national health priority in Australia [2]. Achieving a modest weight loss of 5% of initial body weight is associated with important health benefits [5], including prevention or improved control of type 2 diabetes [6–9], reduced cardiovascular disease risk (CVD) [6, 10] and improvements in other existing health conditions [5], including kidney disease [11] and sleep apnea [12].

Diet quality may influence the development of overweight and obesity. Diet quality refers to the nutritional adequacy of a diet [13], measured by evaluating how closely food patterns align with national dietary guidelines and how varied healthy food choices are within core nutrient-dense food groups [14]. A recent systematic review of prospective cohort studies found a strong association between poor diet quality and greater weight gain [15] in both men and women. For example, in a cohort of 2,245 adult men and women, those who achieved a lower Diet Quality Index gained more weight over eight years compared to those with higher scores [16]. In addition, several studies have found that higher diet quality is inversely related to chronic disease risk, all-cause mortality and cause-specific mortality [17, 18], including CVD and cancer risk.

The role that modifying diet quality plays in assisting overweight and obese individuals to lose weight and maintain weight loss is less clear. To our knowledge, only one previous randomized controlled trial (RCT) has evaluated diet quality changes during a weight loss intervention [19]. The study used the Healthy Eating Index-2005 (HEI-2005) and found the diet quality of women (n = 66) significantly improved (HEI-2005 score 53.9 vs. 57.4, P = 0.002) from baseline to post-intervention after a 16-week behavioral weight loss program. Furthermore, participants with a weight loss of ≥5% had a significantly greater improvement in their diet quality score compared to those with <5% weight loss [19]. This study was a secondary analysis of an RCT with participants randomized to one of two behavioral weight loss programs. The authors did not compare diet quality between the two groups. Data from both groups were combined for the analysis as no significant difference in weight loss was demonstrated between the two treatment groups. Consequently, it is not known whether a weight loss intervention improves the quality of dietary intake relative to controls, nor if different approaches to delivery of weight loss interventions influence diet quality.

The primary aim of this study was to compare changes in diet quality, assessed using the Australian Recommended Food Score (ARFS) [15], in overweight and obese adults randomized to a basic or enhanced version of a commercial web-based weight loss program or a wait-list control group for 12 weeks. The secondary aim was to determine whether there was an association between the extent of weight loss and diet quality at 12 weeks. It was hypothesized that diet quality would improve during the weight loss program in intervention participants and that a higher diet quality score at 12 weeks would be associated with a greater percentage weight loss. We also hypothesized that the enhanced intervention group receiving specific feedback on dietary intake would have greater improvements in diet quality compared to the basic intervention and control groups.

## Methods

### Study design and participants

The prospective RCT recruited overweight and obese adults (n = 309) from the Hunter Region of New South Wales (NSW), Australia, from October to December 2009 [16]. Participants were randomized to one of three groups for 12 weeks: a wait-list control, or a basic or enhanced web-based weight loss intervention [13, 14, 16]. Inclusion criteria included: aged 18 to 60 years, body mass index (BMI) 25 to 40 kg/m^2^, had agreed not to participate in other weight loss programs, had passed a health-screening questionnaire [17], were available for in-person assessments and had access to a computer with email and Internet services. Participants were excluded if they were currently pregnant or trying to conceive, had major medical problems or orthopedic problems, had lost ≥4.5 kg in the previous six months or were taking medications that may affect or be affected by weight loss [14, 16]. The detailed methods have been published elsewhere [16]. The University of Newcastle’s Human Research Ethics Committee approved the study protocol (reference number H-2009-0245) and all participants provided written informed consent [16].

### Interventions

The wait-list control group were not given access to the study website, were asked to maintain their usual dietary intake and physical activity habits and refrain from participating in any other weight loss program during the 12-weeks [16]. Participants in the basic and enhanced intervention groups received free access to the study website, a commercial web-based weight loss program (http://www.biggestloserclub.com.au) provided by SP Health Co Pty Ltd. The key features of the basic and enhanced programs are detailed elsewhere [16]. Conceptually, the weight loss intervention model was based on social cognitive theory [18], targeting self-efficacy, goal setting and self-monitoring of weight, dietary intake and physical activity levels.

### Measures

All measurements from the primary study are detailed elsewhere [16]. Assessors were blinded to participant group allocation. Participant assessments occurred at baseline and at 12-weeks at the University of Newcastle, NSW, Australia. Baseline socio-demographic data were collected by questionnaire. Height was measured using the stretch stature method on a Harpenden portable stadiometer (Holtain Limited, Dyfed, Britain), weight was measured on a digital scale to 0.01 kg (CH-150kp, A&D Mercury Pty Ltd, Australia) and BMI was calculated from height and weight [16].

### Assessment of dietary intake

Dietary intake was assessed using the Australian Eating Survey (AES) administered at baseline and 12 weeks. The AES is a 120-item semi-quantitative food frequency questionnaire (FFQ) and has demonstrated acceptable reliability and relative validity for ranking nutrient intakes in Australian adults [14, 19]. Participants self-reported the frequency of food consumption for the previous 6 months at baseline and for the previous 12 weeks at follow-up. Frequency options for most items ranged from ‘Never’ to ‘4 or more times per day’ for food items and ‘7 or more glasses per day’ for beverages, but varied depending on the item. Nineteen questions relate to the intake of vegetables and 11 items to fruit, with a separate section for seasonal fruit.

The completed AES FFQs were scanned and nutrient intakes computed using FoodWorks (Version 3.02.581) utilizing the Australian food composition database, AusNut 1999 (All Foods) Revision 14 (Australian Government Publishing Service, Canberra) to generate individual mean daily macro- and micronutrient intakes [19].

### The Australian Recommended Food Score (ARFS)

Baseline and 12-week diet quality scores (ARFS) were calculated. The ARFS is a validated measure of diet quality derived from the AES FFQ [19] and is described in detail elsewhere [15, 20]. The ARFS focuses on dietary variety within nutrient-dense, core food groups. The score is calculated based on items listed in the AES FFQ consistent with national recommendations in the Australian Dietary Guidelines [21] and the core foods within the Australian Guide to Healthy Eating [22]. The ARFS score ranges from zero to 73, with eight subscales; Vegetable (0–21), Fruit (0–12), Meat (0–7), Vegetarian Alternatives (0–6), Wholegrain (0–13), Dairy (0–11), Water (0–1) and Condiments (0–2). Most foods within the sub-scales are given one point for a consumption frequency ≥ once per week. A higher total score indicates greater dietary variety within food groups and therefore alignment with national dietary recommendations.

### Statistical analysis

Baseline AES FFQs were completed by 98% of study participants (n = 304). Of these, n = 15 were excluded from the analysis due to missing baseline ARFS values, leaving a total of n = 289 participants with complete dietary intake data at baseline. Shapiro Wilks Goodness of Fit tests were conducted to assess for normality. Descriptive statistics (mean [standard deviation (SD)] for continuous variables and percentages for categorical variables) were used to describe participants’ characteristics at enrolment. Differences between groups at baseline were tested using analysis of variance (ANOVA) for continuous variables and Pearson’s chi-squared test for categorical variables.

Intention-to-treat analysis (ITT), using baseline observations carried forward (BOCF), was used for those lost to follow-up (n = 47) or with missing ARFS values at 12-weeks (n = 22). ANOVA was used to test for changes in diet quality within groups and changes in diet quality between groups. *Post hoc* analysis was performed using Tukey-Kramar HSD or Fishers Exact method to test the difference between groups. Paired t-tests were used to analyze differences within groups, from baseline to 12-weeks. Results are described as means and standard deviations (mean ± SD). Separate multiple linear regression models were used to assess the relationship between diet quality scores (ARFS and individual subscales) at 12 weeks and percentage weight loss. Each model was adjusted for total energy intake at 12 weeks, age, gender, education and weekly household income. Data were considered statistically significant when *P* value <0.05. Data were analyzed using JMP software® (v. 9.0.0, 2010, SAS Institute Inc. Cary, NC).

## Results

### Participant characteristics

There were no significant differences in participant characteristics between groups at baseline (Table 1). Participants (n = 289) were predominantly women (58.5%), Australian born (91%), had not attained a university degree (65.1%), had a weekly household income level of at least $1500 (70.9%) and a mean age of 41.6 ± 10.2 years (Table 1).

**Table 1:** **Baseline demographic characteristics of overweight/obese adults (n = 289) randomized in a commercial web-based weight loss intervention** ^**a**^

	Control n = 97	Basic n = 94	Enhanced n = 98
**Characteristic**	**Mean ± Standard Deviation**		
Age (years)	41.4 ± 9.3	41.8 ± 11.1	41.6 ± 10.3
Weight (kg)	93.8 ± 13.9	95.3 ± 15.2	93.5 ± 14.5
BMI (kg/m^2^)^b^	32.2 ± 3.9	32.4 ± 3.6	32.4 ± 4.3
Height (m)	1.7 ± 0.1	1.7 ± 0.1	1.7 ± 0.1
	**N (%)** ^**c**^		
Gender (Women)	56 (57.7)	55 (58.5)	58 (59.2)
Born in Australia	86 (88.7)	86 (91.5)	91 (92.9)
**Education level**			
< University degree	60 (61.9)	61 (64.9)	67 (68.4)
≥ University degree	37 (38.1)	33 (35.1)	31 (31.6)
**WHI** ^**d**^			
< $1500	29 (29.9)	31 (33.0)	24 (24.5)
≥ $1500	64 (66.0)	56 (59.6)	68 (69.4)
Did not know/answer	3 (3.1)	3 (3.2)	3 (3.1)

^a^No significant difference between groups at baseline.

^b^BMI = Body Mass Index.

^c^N (%) Column number of subjects and percentage within demographic category.

^d^WHI = Weekly Household Income.

### ARFS at baseline

The baseline ARFS and subscales, and daily energy and macronutrient intake, are reported by group in Table 2. Energy and macronutrient intake at baseline did not differ between study groups. Also, no significant differences were found between the study groups for ARFS or any subscales. The mean ARFS for all participants at baseline was 33.4 ± 9.7 (range 3 to 56) out of a maximum possible score of 73. Only 3.8% (n = 11) achieved an ARFS score ≥50, with 28% (n = 81) obtaining a score ≥40.

**Table 2:** **Baseline and 12-week change in weight, energy, macronutrient, and diet quality scores in overweight/obese adults (n = 289)**

	Baseline ^a^			Change from Baseline			Difference between groups for change from Baseline
	Control	Basic	Enhanced	Control	Basic	Enhanced	
Variable	Mean ± Standard Deviation						P Value
Weight (kg)	93.8 ± 13.9	95.3 ± 15.2	93.5 ± 13.5	0.4 ± 2.4	-2.2 ± 3.4^#^	-3.0 ± 4.0^#^	<0.001
Weight (%)				0.4 ± 2.5	-2.3 ± 3.6^#^	-3.3 ± 4.3^#^	<0.001
BMI (kg/m^2^)^b^	32.2 ± 3.9	32.4 ± 3.6	32.4 ± 4.30	0.1 ± 0.1	-0.7 ± 0.1^#^	-1.0 ± 0.1^#^	<0.001
**Energy (kJ/day)**	10,409 ± 3,287	9,982 ± 3,201	10,260 ± 3,288	-865.4 ± 2,260	-1,032 ± 2,519	-1,367 ± 2,204	0.31
Protein (%)	18.2 ± 3.2	18.7 ± 3.30	18.5 ± 3.30	1.0 ± 2.5	1.1 ± 2.7	1.4 ± 2.7	0.56
CHO (%)^c^	46.2 ± 6.7	45.2 ± 6.50	46.1 ± 7.80	-1.0 ± 4.7	-0.5 ± 4.0	-0.6 ± 4.7	0.74
Fat (%)	31.3 ± 5.0	31.3 ± 5.30	32.0 ± 5.50	-0.2 ± 4.0	-0.6 ± 0.4	-1.4 ± 4.6	0.12
Sat Fat (%)^d^	13.4 ± 2.7	13.4 ± 2.80	13.9 ± 2.70	-0.4 ± 2.1	-0.6 ± 2.3	-1.1 ± 2.6	0.17
Fiber (g/day)	27.7 ± 9.0	27.7 ± 8.70	28.7 ± 9.60	-2.0 ± 6.5	-0.6 ± 7.3	-1.1 ± 6.3	0.34
**Total ARFS** ^**e**^	32.6 ± 9.0	33.9 ± 10.3	33.8 ± 9.90	0.1 ± 6.6	0.2 ± 5.5	2.2 ± 5.7^#^	<0.05
Vegetable	12.7 ± 4.5	12.8 ± 4.60	12.9 ± 4.30	-0.2 ± 3.7	0.0 ± 2.4	0.5 ± 2.7	0.26
Fruit	4.8 ± 2.9	5.0 ± 3.2	5.1 ± 3.1	-0.1 ± 2.2	0.1 ± 1.8	0.6 ± 2.4	0.08
Meat	2.7 ± 1.4	2.7 ± 1.3	2.7 ± 2.7	0.3 ± 1.2	0.2 ± 1.1	0.5 ± 1.1	0.31
Vegetarian Alt^f^	1.7 ± 1.2	1.8 ± 1.3	1.7 ± 1.2	-0.1 ± 1.1	0.0 ± 0.9	0.0 ± 1.0	0.88
Wholegrain	5.1 ± 2.1	5.2 ± 2.0	5.1 ± 2.0	-0.1 ± 1.7	0.0 ± 1.4	0.3 ± 1.4	0.09
Dairy	4.1 ± 1.7	4.6 ± 2.0	4.6 ± 1.7	0.1 ± 1.5	-0.1 ± 1.5	0.2 ± 1.4	0.26
Water	0.4 ± 0.5	0.5 ± 0.5	0.5 ± 0.5	0.1 ± 0.4	0.1 ± 0.4	0.2 ± 0.5	0.61
Condiments	1.0 ± 0.7	1.3 ± 0.8	1.2 ± 0.8	0.0 ± 0.7	-0.1 ± 0.6	-0.1 ± 0.7	0.26

^a^No significant difference among groups at baseline.

^b^BMI = Body Mass Index.

^c^CHO = Carbohydrate.

^d^Sat Fat = Saturated Fat.

^e^ARFS = Australian Recommended Food Score.

^f^Vegetarian Alt = Vegetarian Alternatives.

^#^Statistically significant from the change in the control group.

### Differences in ARFS change from baseline to 12 weeks between groups

The enhanced and basic groups (-3.0 ± 4.0 kg or -3.3% and -2.2 ± 3.4 kg or -2.3%, respectively) lost significantly more weight than the control group (0.4 ± 2.4 kg or 0.4% *P* < 0.001) with no difference between the enhanced and basic groups (Table 2). There were no statistically significant differences between the study groups in change in total energy intake, protein, carbohydrate, fat or saturated fat as a percentage of total energy, or fiber intake (Table 2). The mean change in ARFS in the enhanced group (2.2 ± 5.7) was significantly greater (*P* = 0.03) than the control group whereas there was no difference between the basic and control groups. Change from baseline was not significantly different between the study groups for the ARFS subscales (Table 2).

### Within group changes in ARFS from baseline to 12 weeks

Aspects of diet quality improved within each study group from baseline to 12 weeks (Table 3). The ARFS (*t*(3.9), *P* < 0.001), and the fruit (*t*(2.4), *P* = 0.02), meat (*t*(4.3) *P* < 0.001), wholegrain (*t*(2.3), *P* = 0.02), and water (*t*(3.9), *P* < 0.001) subscales improved significantly from baseline in the enhanced group. The total ARFS did not change significantly in the basic or control group, but the meat (*t*(2.0) and *t*(2.5), <0.05) and water (*t*(3.3) and *t*(2.8), *P* ≤ 0.01) subscales increased significantly.

**Table 3:** **Paired t-test results for change in diet quality scores from baseline to 12-weeks within study groups of overweight/obese adults (n = 289) randomized to a wait-list control, basic or enhanced version of a commercial web-based weight loss intervention**

	Control n = 97		Basic n = 94		Enhanced n = 98	
Variable	Mean	***t***-ratio	Mean	***t***-ratio	Mean	***t***-ratio
Total ARFS^a^	0.06	0.09	0.23	0.41	2.21	3.88***
Vegetable	-0.22	-0.57	0.03	0.13	0.48	1.75
Fruit	-0.09	-0.41	0.13	0.68	0.58	2.45*
Meat	0.29	2.46*	0.23	1.99*	0.47	4.32***
Vegetarian Alt^b^	-0.06	-0.54	-0.01	-0.11	0.01	0.10
Wholegrain	-0.11	-0.65	0.03	0.22	0.34	2.30*
Dairy	0.12	0.82	-0.11	-0.68	0.23	1.69
Water	0.12	2.77**	0.14	3.32**	0.18	3.94***
Condiments	0.01	0.15	-0.15	-2.46*	-0.06	-0.86

^a^ARFS = Australian Recommended Food Score.

^b^Vegetarian Alt = Vegetarian Alternatives.

*Statistically significantly difference in baseline and 12-week value within groups P < 0.05.

**Statistically significantly difference in baseline and 12-week value within groups P < 0.01.

***Statistically significantly difference in baseline and 12-week value within groups P < 0.001.

### Relationship between diet quality score and weight loss at 12 weeks

The results of the multiple linear regression models are shown in Table 4. In the total sample (n = 289), improvements in the ARFS and the fruit, meat, wholegrain, dairy and water subscale scores at 12 weeks were significantly associated with greater percentage weight loss. Further, we can estimate that weight loss increased by an average 0.1% when the ARFS increased by one point.

**Table 4:** **The relationship between diet quality score and percentage weight loss of overweight/obese adults (n = 289)**

Variable	β ^a^	SE ^b^	95% CI ^c^	***r*** ^***d***^	***P***Value
Total ARFS^e^	0.10	0.03	0.044, 0.152	0.22	<0.001
Vegetable	0.11	0.06	-0.003, 0.221	0.12	0.06
Fruit	0.18	0.09	0.013, 0.352	0.13	0.04
Meat	0.66	0.18	0.308, 1.013	0.22	<0.001
Vegetarian Alt^f^	0.17	0.20	-0.232, 0.569	0.05	0.41
Wholegrain	0.43	0.13	0.167, 0.691	0.20	0.001
Dairy	0.29	0.14	0.020, 0.566	0.13	0.04
Water	1.69	0.48	0.743, 2.643	0.21	<0.001
Condiments	-0.24	0.32	-0.872, 0.399	-0.05	0.47

Dependent Variable: Percentage Weight loss.

Models were adjusted for confounders: total energy intake at 12-weeks, age, gender, education and weekly household income.

^a^β = Regression coefficient.

^b^SE = Standard Error.

^c^CI = Confidence Interval.

^*d*^ *r =* Partial Correlation coefficient.

^e^ARFS = Australian Recommended Food Score.

^f^Vegetarian Alt = Vegetarian Alternatives.

## Discussion

The primary aim of this study was to compare changes in diet quality in an online RCT with two online treatment arms of varying intensity during a 12-week web-based weight loss program. A secondary aim was to determine whether there was an association between diet quality score at 12 weeks and percentage weight loss. The change in overall diet quality in the enhanced group was significantly greater than the control group. However, there were no significant differences between the enhanced and basic, or basic and control groups. Importantly, improvements in diet quality scores were associated with a greater percentage weight loss, regardless of treatment group.

No significant differences were found in change in ARFS subscales from baseline to 12 weeks between study groups, however two ARFS subscales improved significantly within all study groups over this time. Improvements in the fruit, meat, wholegrain and water subscales were seen in the enhanced group, while the meat and water subscales improved in both the basic and control groups. There was no improvement in the vegetable subscale. It was hypothesized that improvements in diet quality in the enhanced group would be due to the increased variety in fruit and vegetables, as a result of the additional feedback the group received regarding consumption of these foods. Similar results were found in Webber & Lee’s (2011) study, where the increase in fruit score was significant (*P* < 0.05) and the increase in vegetable score was not [23]. A study by Booth *et al*. (2008) suggested that different food types might have different adjustment periods. For example, improvement in fruit intake can be achieved fairly quickly, compared to an increase in vegetable intake which may require a longer time period to achieve [24]. This suggests that vegetable intake may be more difficult to improve in a 12-week study. Additional strategies other than feedback may be required to improve the variety of vegetable intake.

The present study found that higher diet quality scores at 12 weeks were associated with greater percentage weight loss (as diet quality scores increased, percentage weight loss increased). This is important, as previous studies examining the relationship between diet quality and weight change have primarily focused on weight gain [25–27]. Also, a focus on improvement in diet quality can be incorporated within dietary intervention goals. Our findings are consistent with the study by Webber & Lee (2011), which found that participants with a weight loss of ≥5% had a significantly greater improvement in diet quality score compared to those with <5% weight loss [23].

From the current study findings, weight loss increased by an average of 1% of body weight when the ARFS increased by ten points (or 0.1% for each one point increase). In simplified terms, an increase of one ARFS point equates to consuming one new/different food at least once per week. Also we demonstrated for some ARFS sub-scales (e.g. meat, wholegrain and water) that a one-point increase in score increased weight loss (0.7% meat, 0.4% wholegrain and 1.7% water). At a population level, a 0.1% decrease in weight may be important. We have demonstrated that an enhanced web-based weight loss intervention can significantly improve diet quality (i.e. mean increase in ARFS of 2.2 in 12 weeks). Therefore, further investigation of dietary and/or weight loss interventions that can be implemented at the population level to improve diet quality are warranted.

Analysis of the association between ARFS subscales at 12-weeks and percentage weight loss found several significant positive associations, including fruit, meat, wholegrain, dairy and water subscales. This suggests that as participants consumed a greater variety of foods from within the fruit, meat, wholegrain, dairy or water sub-scales on a regular basis (i.e. ≥ once per week) the amount of weight loss increases, independent to their total energy intake. For example, they may have increased their intake of breakfast cereals, oats, rice, wholegrain bread, noodles or pasta to ≥ once per week each to increase their score in the wholegrain subscale. Several studies have found higher intakes of these foods are associated with weight loss [28–39]. However, these studies usually measure quantity rather than variety of consumption. Studies found that higher intakes of fruit, protein and high-fiber breads and cereals were associated with greater weight loss [28–30, 39]. Higher intakes of fruit and wholegrain were also shown to be a significant predictor of weight loss [31, 32]. Several studies suggest there is an association between higher dairy intake and greater weight loss [33–35, 38], however some studies are inconclusive [35, 40] and others have found that high intakes of dairy to lead to weight gain [41, 42]. Studies have also shown that increasing water consumption during weight loss interventions can lead to greater weight loss [43, 44]. Therefore, it may be effective to focus on these particular food groups during weight loss interventions to facilitate greater weight loss. Further studies examining the association between these food groups, in the context of diet quality and weight loss during weight loss interventions are needed to support these findings. Consumption of a wide variety of nutrient-dense foods, thereby improving diet quality, may lead to greater weight loss. Using a brief tool such as the ARFS to measure diet quality would carry a lower respondent burden than completing an FFQ. Diet quality tools only include foods that are consistent with national dietary guidelines, so have a smaller number of items than an FFQ, and fewer frequency categories [15].

A limitation of this study is the use of a self-reported tool to assess dietary intake. There is also potential bias from the training effect of the FFQ and under-reporting, common among overweight and obese individuals [45, 46]. In addition, participation in the intervention may have influenced dietary recall, making participants more aware of their eating patterns. Another limitation is the potential low sensitivity for scoring given that having a recommended food once per week adds one point to the total score in the same way as having the same food three or more times per week [15]. Moreover, the ARFS does not reflect energy-dense nutrient-poor foods. However, the FFQ and the ARFS are both validated reliable tools to assess dietary intakes in Australian adults [19].

Another limitation is in respect to generalizability. The study sample included a higher proportion of high-income earners (those with a weekly household income > AUS$1500 were regarded as having ‘high incomes’ at baseline [47]), university educated individuals, and those born in Australia, compared to the Australian population. Therefore, the results may not be applicable to other ethnic groups and those of lower-socioeconomic status. However, the study recruited almost equal proportions of male and females, which is unique, as the majority of weight loss trials recruit predominantly females [48]. Also, this was a secondary analysis of an RCT powered to detect differences in BMI change between groups. Therefore, the magnitude and significance of the difference between the groups of the measures in this analysis may have been limited by the sample size. Consequently, additional investigation is required with a larger sample size, powered to detect a significant difference in diet quality. The main strength of the study is that this is only the second study to explore changes in diet quality as a result of participating in a weight loss intervention. Additional strengths include the RCT design and use of ITT analysis using BOCF.

## Conclusions

Significant improvements in diet quality were detected in the enhanced intervention group, from baseline to 12 weeks, compared to control. In addition, higher diet quality scores at 12 weeks were associated with greater percentage weight loss. However, despite improvements, diet quality scores remained low in the enhanced group. This study highlights the importance of addressing diet quality in weight loss interventions and that a brief diet tool can be applied to measure diet quality as part of a weight loss intervention. The scoring method of a diet quality index suggests it is a simple tool to evaluate diet in the context of weight loss and identify areas for improvement.

## References

[1] Swinburn BA, Sacks G, Hall KD, McPherson K, Finegood DT, Moodie ML, Gortmaker SL (2011). The global obesity pandemic: shaped by global drivers and local environments. Lancet.

[2] AIHW (2004). The relationship between overweight, obesity and cardiovascular disease: a literature review prepared for the National Heart Foundation Australia.

[3] AIHW (2010). Australia's health 2010.

[4] Bassuk SS, Manson JE, Editor-in-Chief: KH: **Obesity/Overweight: Health Consequences of.** In *International Encyclopedia of Public Health*. Oxford: Academic; 2008:589–602.

[5] NHMRC (2012). Management of Overweight and Obesity in Adults, Adolescents and Children. Clinical Practice Guidelines for Primary Care Health Professionals. Public Consultation Draft.

[6] Hamman RF, Wing RR, Edelstein SL, Lachin JM, Bray GA, Delahanty L, Hoskin M, Kriska AM, Mayer-Davis EJ, Pi-Sunyer X, Regensteiner J, Venditti B, Wylie-Rosett J (2006). Effect of weight loss with lifestyle intervention on risk of diabetes. Diabetes Care.

[7] Galani C, Schneider H (2007). Prevention and treatment of obesity with lifestyle interventions: review and meta-analysis. Int J Pub Health.

[8] Norris SL, Zhang X, Avenell A, Gregg E, Schmid CH, Lau J (2005). Long-term non-pharmacological weight loss interventions for adults with prediabetes. Cochrane Database Syst Rev.

[9] Dale KS, Mann JI, McAuley KA, Williams SM, Farmer VL (2009). Sustainability of lifestyle changes following an intensive lifestyle intervention in insulin resistant adults: follow-up at 2-years. Asia Pac J Clin Nutr.

[10] Groeneveld IF, Proper KI, van der Beek AJ, van Mechelen W (2010). Sustained body weight reduction by an individual-based lifestyle intervention for workers in the construction industry at risk for cardiovascular disease: results of a randomized controlled trial. Prev Med.

[11] Navaneethan SD, Yehnert H, Moustarah F, Schreiber MJ, Schauer PR, Beddhu S (2009). Weight loss interventions in chronic kidney disease: a systematic review and meta-analysis. Clin J Am Soc Nephrol.

[12] Tuomilehto HP, Seppa JM, Partinen MM, Peltonen M, Gylling H, Tuomilehto JO, Vanninen EJ, Kokkarinen J, Sahlman JK, Martikainen T, Soini EJ, Randell J, Tukiainen H, Uusitupa M, Kuopio Sleep Apnea Group (2009). Lifestyle intervention with weight reduction: first-line treatment in mild obstructive sleep apnea. Am J Respir Crit Care Med.

[13] Collins CE, Morgan PJ, Jones P, Fletcher K, Martin J, Aguiar EJ, Lucas A, Neve MJ, Callister R (2012). A 12-week commercial web-based weight-loss program for overweight and obese adults: randomized controlled trial comparing basic versus enhanced features. J Med Internet Res.

[14] Hutchesson MJ, Collins CE, Morgan PJ, Watson JF, Guest M, Callister R (2013). Changes to dietary intake during a 12-week commercial web-based weight loss program: a randomized controlled trial. Eur J Clin Nutr.

[15] Collins CE, Young AF, Hodge A (2008). Diet quality is associated with higher nutrient intake and self-rated health in mid-aged women. J Am Coll Nutr.

[16] Collins CE, Morgan PJ, Jones P, Fletcher K, Martin J, Aguiar EJ, Lucas A, Neve M, McElduff P, Callister R (2010). Evaluation of a commercial web-based weight loss and weight loss maintenance program in overweight and obese adults: a randomizedcontrolled trial. BMC Public Health.

[17] Not a journal article. Replace "Norton K (2005). Sports Medicine Australia pre-exercise screening system. Austr Dep Health Ageing 2005." with "Norton K.

[18] Bandura A (1986). Social foundations of thought and action: A Social Cognitive Theory.

[19] Collins C, Boggess M, Watson J, Guest M, Duncanson K, Pezdirc K, Rollo M, MJ H, Burrows T (2013). Reproducibility and comparative validity of a food frequency questionnaire for Australian adults. Clin Nutr.

[20] Marshall S, Watson J, Burrows T, Guest M, Collins C (2012). The development and evaluation of the Australian child and adolescent recommended food score: a cross-sectional study. Nutr J.

[21] NHMRC (2013). Australian Dietary Guidelines.

[22] NHMRC (2013). The Australian Guide to Healthy Eating.

[23] Webber KH, Lee E (2011). The diet quality of adult women participating in a behavioural weight-loss programme. J Hum Nutr Diet.

[24] Booth A, Nowson C, Worsley A, Margerison C, Jorna M (2008). Dietary approaches for weight loss with increased intakes of fruit, vegetables and dairy products. Nutr Diet.

[25] Quatromoni PA, Pencina M, Cobain MR, Jacques PF, D'Agostino RB (2006). Dietary quality predicts adult weight gain: findings from the Framingham offspring study. Obesity.

[26] Romaguera D, Norat T, Vergnaud A, Mouw T, May AM, Agudo A, Buckland G, Slimani N, Rinaldi S, Couto E, Clavel-Chapelon F, Boutron-Ruault MC, Cottet V, Rohrmann S, Teucher B, Bergmann M, Boeing H, Tjonneland A, Halkjaer J, Jakobsen MU, Dahm CC, Travier N, Rodriguez L, Sanchez MJ, Amiano P, Barricarte A, Huerta JM, Luan J, Wareham N, Key TJ (2010). Mediterranean dietary patterns and prospective weight change in participants of the EPIC-PANACEA project. Am J Clin Nutr.

[27] Wolongevicz DM, Zhu L, Pencina MJ, Kimokoti RW, Newby PK, D'Agostino RB, Millen BE (2010). Diet quality and obesity in women: the Framingham NUTRITION STUDIES. Br J Nutr.

[28] Noakes M, Keogh JB, Foster PR, Clifton PM (2005). Effect of an energy-restricted, high-protein, low-fat diet relative to a conventional high-carbohydrate, low-fat diet on weight loss, body composition, nutritional status, and markers of cardiovascular health in obese women. Am J Clin Nutr.

[29] Sartorelli DS, Franco LJ, Cardoso MA (2008). High intake of fruits and vegetables predicts weight loss in Brazilian overweight adults. Nutr Res.

[30] Leidy HJ, Carnell NS, Mattes RD, Campbell WW (2007). Higher protein intake preserves lean mass and satiety with weight loss in pre-obese and obese women. Obesity.

[31] Schroder KEE (2010). Effects of fruit consumption on body mass index and weight loss in a sample of overweight and obese dieters enrolled in a weight-loss intervention trial. Nutrition.

[32] Schulz M, Kroke A, Liese AD, Hoffmann K, Bergmann MM, Boeing H (2002). Food groups as predictors for short-term weight changes in men and women of the EPIC-Potsdam cohort. J Nutr.

[33] Faghih S, Abadi AR, Hedayati M, Kimiagar SM (2011). Comparison of the effects of cows' milk, fortified soy milk, and calcium supplement on weight and fat loss in premenopausal overweight and obese women. Nutr Metab Cardiovasc Dis.

[34] Zemel MB, Teegarden D, Loan MV, Schoeller DA, Matkovic V, Lyle RM, Craig BA (2009). Dairy-rich diets augment fat loss on an energy-restricted diet: a multicenter trial. Nutrients.

[35] Zemel MB, Richards J, Milstead A, Campbell P (2005). Effects of calcium and dairy on body composition and weight loss in African-American adults. Obes Res.

[36] Zemel MB, Richards J, Mathis S, Milstead A, Gebhardt L, Silva E (2005). Dairy augmentation of total and central fat loss in obese subjects. Int J Obes (Lond).

[37] Zemel MB, Thompson W, Milstead A, Morris K, Campbell P (2004). Calcium and dairy acceleration of weight and fat loss during energy restriction in obese adults. Obes Res.

[38] Wyatt HR, Jortberg BT, Babbel C, Garner S, Fang D, Grunwald GK, Hill JO (2008). Weight loss in a community initiative that promotes decreased energy intake and increased physical activity and dairy consumption: calcium weighs-in. J Phys Act Health.

[39] Layman DK, Evans EM, Erickson D, Seyler J, Weber J, Bagshaw D, Griel A, Psota T, Kris-Etherton P (2009). A moderate-protein diet produces sustained weight loss and long-term changes in body composition and blood lipids in obese adults. J Nutr.

[40] Palacios C, Bertran JJ, Rios RE, Soltero S (2011). No effects of low and high consumption of dairy products and calcium supplements on body composition and serum lipids in Puerto Rican obese adults. Nutrition.

[41] Bowen J, Noakes M, Clifton PM (2005). Effect of calcium and dairy foods in high protein, energy-restricted diets on weight loss and metabolic parameters in overweight adults. Int J Obes (Lond).

[42] Barr SI, McCarron DA, Heaney RP, Dawson-Hughes B, Berga SL, Stern JS, Oparil S (2000). Effects of increased consumption of fluid milk on energy and nutrient intake, body weight, and cardiovascular risk factors in healthy older adults. J Am Diet Assoc.

[43] Stookey JD (2010). Drinking water and weight management. Nutr Today.

[44] Dennis EA, Dengo AL, Comber DL, Flack KD, Savla J, Davy KP, Davy BM (2010). Water consumption increases weight loss during a hypocaloric diet intervention in middle-aged and older adults. Obesity (Silver Spring, Md).

[45] Macdiarmid J, Blundell J (1998). Assessing dietary intake: who, what and why of under-reporting. Nutr Res Rev.

[46] Tooze JA, Subar AF, Thompson FE, Troiano R, Schatzkin A, Kipnis V (2004). Psychosocial predictors of energy underreporting in a large doubly labeled water study. Am J Clin Nutr.

[47] ABS (2009). Household income and income distribution 2007–08.

[48] Pagoto SL, Schneider KL, Oleski JL, Luciani JM, Bodenlos JS, Whited MC (2012). Male inclusion in randomized controlled trials of lifestyle weight loss interventions. Obesity (Silver Spring).

